# Supplementary material for: Non-local validated parametrization of an agent-based model of local-scale Taenia solium transmission in North-West Peru
Source: PLoS One. 2022 Sep 27;17(9):e0275247. doi: 10.1371/journal.pone.0275247 (PMC9514638; doi:10.1371/journal.pone.0275247)
Supplement: S1 File — (DOCX) [file pone.0275247.s001.docx]

# Supporting information S1

Demographic module

This supplementary material details data and methods related to the ABM demographic module, including:

1. Death rates, by age range.
2. Birth rates.
3. Emigration rates, by age range.
4. Immigration rates, by age range and origin.
5. Detailed description of the methodology used to model demographic changes in the population using data on births, deaths and human movements.

# Death rates, by age range

Country level and survey-specific data can provide insights on ‘natural’ population changes. Overall deaths, by age range, can be deduced from country-level data [1] and Piura specific data [2], using information on the region an individual lived in 5 years ago, combined with [3] for regional population by age range in 2016 and 2011).

Within-country movement is accounted for and not confused with deaths. Emigration out of the country is harder to track, which is why S1 Table 1 does not distinguish it from deaths. Our knowledge of population movements at a more granular level (e.g. province, district, or village-level) is more limited and we were therefore not able to estimate death rates at these levels.

**S1 Table 1**

| **Age range** | 0-4 | 5-9 | 10-14 | 15-19 | 20-24 | 25-29 | 30-34 | 35-39 |
| --- | --- | --- | --- | --- | --- | --- | --- | --- |
| All Peru | 3.1% | 3.4% | 4.6% | 4.3% | 4.1% | 1.8% | 0.4% | 0.5% |
| Piura region | 1.9% | 1.9% | 3.4% | 3.8% | 4.2% | 4.1% | 4.2% | 4.1% |
| Age range | 40-44 | 45-49 | 50-54 | 55-59 | 60-64 | 65-69 | 70-74 | 75+ |
| All Peru | 0.8% | 1.7% | 1.9% | 1.3% | 2.3% | 6.0% | 11.2% | 47.4% |
| Piura region | 4.5% | 4.9% | 5.3% | 6.6% | 9.1% | 13.4% | 19.6% | 42.3% |

*Percentage of Peru and Piura population, by age range, that died or emigrated out of the country between 2011 and 2016. Sources:* [1–3]

# Birth rates

For the 2011 to 2016 period, the average birth rate was: 18.0 per thousand inhabitants per year for Peru as a whole [1], and 20.3 and 23.1 per thousand for the Piura region and the Ayabaca province within the Piura region respectively [2]. Figures for rural Piura for 2012 using the Demographic and Health Survey [3] are similar to those for the Piura region overall (20.9 per thousand inhabitants per year). Data from prior surveys in target villages show similar birth rates, but the precision is lower given the size of the subsample aged one year and younger.

# Emigration rates, by age range

Individuals that emigrate affect the age structure of the village population. Emigration out of the country cannot easily be distinguished from death, but within-country movement can.

There are two sources of information for within-country movement. National surveys [2] provide extensive information as to how many individuals living in a region in a given year were living in the same district 5 years ago. This does not include individuals that changed village within a district. Meanwhile, endemic villages targeted for past studies have sometimes been visited twice, as was the case for the study described in [4] (we will refer to this study as “GATES2” in what follows). This enables us to estimate the rate of movement out of specific endemic villages, excluding emigration to other countries or deaths, using the estimates in S1 Table 2. Given the low samples for higher ages and the fact that we are combining data from two different data sources, data for 60 years and above are not reliable.

Data for Piura (computed over 5 years) are smoother and more reliable than data from the Gates 2 villages (computed over 1 year). Both tables are not fully comparable as S1 Table 2 does not include mobility within a district. However, overall, figures are similar enough (an estimated 10.3% of individuals living in Piura in 2011 had moved by 2016 vs. 12-13% of the Gates 2 village population if we use the yearly emigration rate found in S1 Table 3 for 5 years in a row). Piura data was therefore used for the purpose of the model.

**S1 Table 2: Percentage of Piura population, by age range, that changed district within Peru between 2011 and 2016**

| Age range | 0-4 | 5-9 | 10-14 | 15-19 | 20-24 | 25-29 | 30-34 | 35-39 |
| --- | --- | --- | --- | --- | --- | --- | --- | --- |
| Piura region | 7.3% | 7.1% | 12.8% | 18.1% | 15.3% | 12.6% | 10.7% | 9.2% |
| Age range | 40-44 | 45-49 | 50-54 | 55-59 | 60-64 | 65-69 | 70-74 | 75+ |
| Piura region | 11.2% | 7.6% | 7.4% | 6.8% | 6.5% | 5.7% | 5.2% | 4.2% |

Sources: [2,4,5]

**S1 Table 3: Estimated yearly rate of emigration out of GATES2 villages**

| Age range | 0-4 | 5-9 | 10-14 | 15-19 | 20-24 | 25-29 |
| --- | --- | --- | --- | --- | --- | --- |
| % from village moved within country | 4.2% | 3.1% | 0.5% | 3.9% | 4.7% | 4.3% |
| Age range | 30-34 | 35-39 | 40-44 | 45-49 | 50-54 | 55-59 |
| % from village moved within country | 3.8% | 0.8% | 1.5% | 0.8% | 0.3% | 0.2% |

Movement out of the village of origin but within the country. Not all data was collected at the exact same time, but most data was collected between early 2007 (for the baseline census) and late 2007 (for the second visit). Sources: [4] and S1 Table 1 data

# Immigration rates, by age range and origin

Again, immigration rates are reasonably well-known for the Piura region. In total, the 2016 Piura population that, in 2011, lived in another district than their 2016 district, represented 12.6% of the 2011 Piura population.

Meanwhile, we also have an estimate for the GATES2 study. The immigration rate is 3.3% per year and 17.6% over 5 years, including movement between two villages within the same study. Though this is higher than for the Piura region, figures are similar enough and Piura figures should be more reliable, hence this is what was used in the model.

The origin of these migrants is critical: they may bring new taenia worms into the village and, their infection rate, if very different from that of the village, could significantly affect average village rates. Current estimates are that 61% of immigrants came from urban localities. However, these are based on questionnaires in the control and intervention village field trials [5,6] the GATES2 trial regarding the village of origin of migrants, but the coding of answers appears very unreliable. Field studies are planned to obtain reliable figures.

**S1 Table 4: 2016 Piura population, by age range, that lived in another district or abroad in 2011, as a share of 2011 Piura population in age range**

| Age range | 0-4 | 5-9 | 10-14 | 15-19 | 20-24 | 25-29 | 30-34 | 35-39 |
| --- | --- | --- | --- | --- | --- | --- | --- | --- |
| Piura region | 7.2% | 6.8% | 9.5% | 13.1% | 13.9% | 12.7% | 11.1% | 7.2% |
| Age range | 40-44 | 45-49 | 50-54 | 55-59 | 60-64 | 65-69 | 70-74 | 75+ |
| Piura region | 8.5% | 7.5% | 7.0% | 6.2% | 5.7% | 4.9% | 4.4% | 3.6% |

Sources: [1–3]

**S1 Table 5: Estimated yearly rate of immigration into GATES2 villages**

| Age range | 0-4 | 5-9 | 10-14 | 15-19 | 20-24 | 25-29 | 30-34 | 35-39 |
| --- | --- | --- | --- | --- | --- | --- | --- | --- |
| % in age range that moved into village | 5.2% | 3.9% | 2.4% | 2.6% | 3.7% | 4.7% | 5.5% | 2.5% |
| Age range | 40-44 | 45-49 | 50-54 | 55-59 | 60-64 | 65-69 | 70-74 | 75+ |
| % in age range that moved into village | 2.9% | 2.8% | 2.0% | 2.8% | 0.7% | 1.7% | 1.2% | 1.5% |

*Sources:* [4]

# Methodology used to represent demographic changes

Population changes are connected with deaths, births, and population movements. This section describes how these changes are modelled.

The reality of natural demographic changes (S1 Fig 1) is complex: it includes births and deaths, emigration to different places (low, medium or high risk), and immigration into the village of individuals that often have a complex history (e.g., stays in multiple places with diverse risks, including possibly the village they are now moving into). Short-term travel to various destinations (already included in CystiAgents and not represented on the graph in S1 Fig 1) adds to long-term trends.

**S1 Fig 1: ‘Reality’ of demographic changes**

**
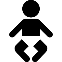

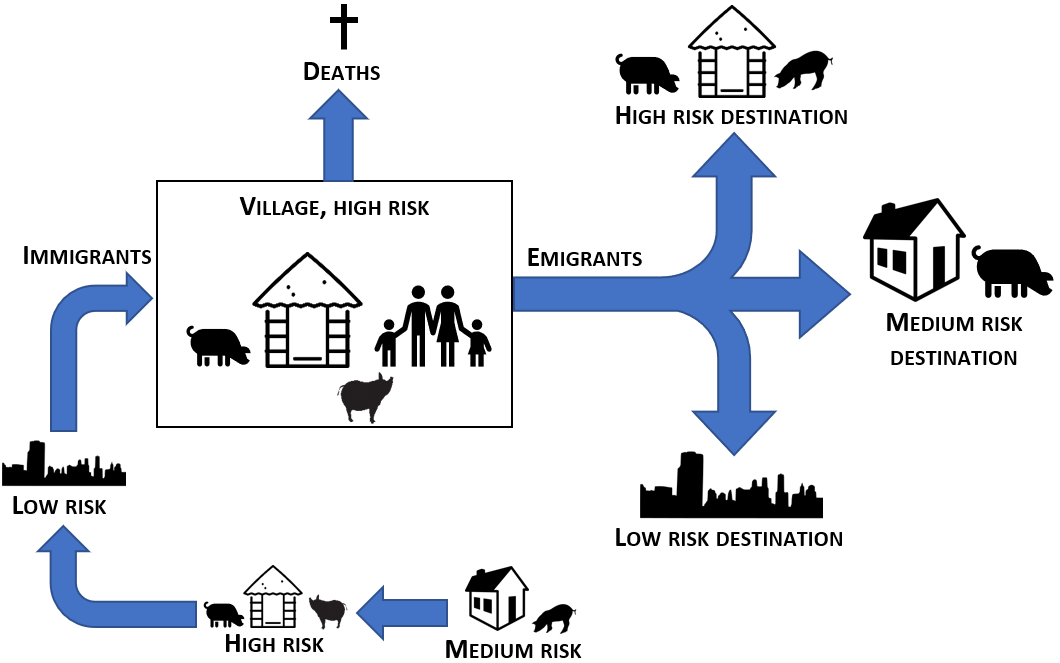
**

**Births**

It is proposed to model natural demographic changes as follows:

Deaths are modelled through a probability of dying applied to each age range. Individuals that die are then removed from the simulation.

Meanwhile, for the purpose of modelling what takes place within the target villages, emigration is equivalent to death, and removing emigrants from the simulation is enough.

Immigrants affect the demographic composition and disease status of the village. For example, should all immigrants come from low-risk areas, they could significantly decrease the overall prevalence rate. The reality of immigration is complex, but we simplify it as follows:

1. Places of origin are modelled solely as “low-risk” or “high-risk” (modelled by assuming individuals living in such a locality face the same risks as in the target villages).
2. Immigration from multiple places with different risk levels is simplified as follows: if N immigrants in a given age-range have spent x% of their past life in high-risk areas and (1-x%) in low-risk areas, they are equated with N * x% immigrants coming from high-risk areas exclusively, and N * (1-x%) coming from low-risk areas (see S1 Fig2). This is acceptable if we can assume that 1) infection risks are mostly additive, 2) high risk locations and low risk locations, for a given age range of immigrants, are randomly distributed.

**S1 Fig2: Simplification of people’s history when they have lived in different places with various levels of risks before coming to the village**


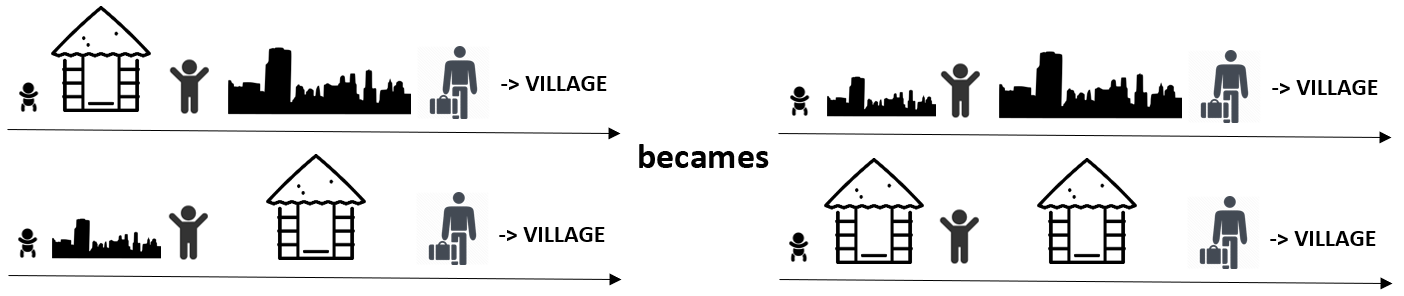


Finally, births bring new inhabitants to the village with no infection status and age 0. The simplified demographic model therefore looks as follows:

**S1 Fig3: Simplified model of demographic changes**


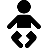

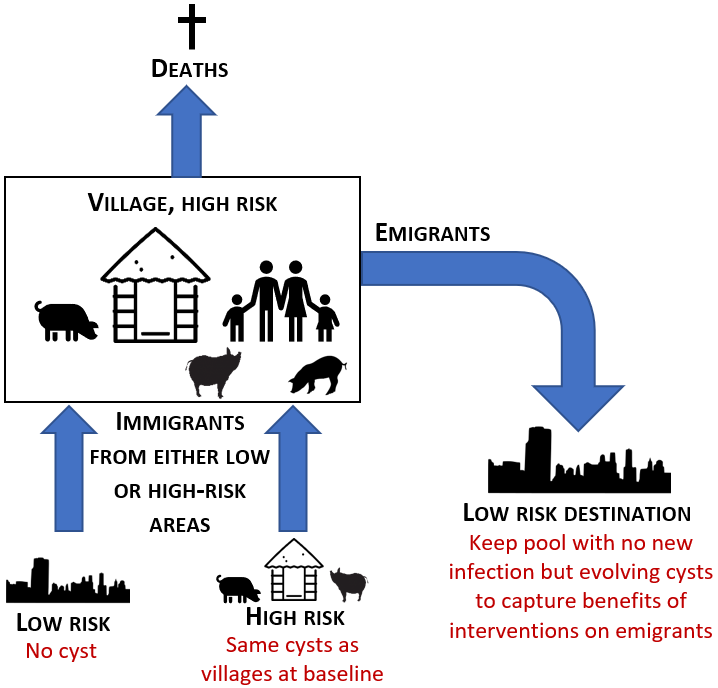


**Births**

# Population growth

With the actual value of births, deaths, and migrations, it is expected that the population of the Piura region will grow by around 0.82% per year (based on INEI data for 2011 and 2016 [7]). Over 85 years of simulation, the population of the region would double. This is a problem for modelling, as if the model’s population is allowed to grow, either household size will reach unrealistic levels or new households will have to be created and located within growing village boundaries, in line with local practices (e.g., subdivision of the parents’ holding), which could prove complex. Further, using current birth and death rates over a 85 years’ period may significantly change the age structure of the population.

Instead, we propose to tweak the current values of birth, death, emigration and immigration rates so that: i) population by age range remain constant, ii) overall numbers of emigrants, births, deaths and immigrants (total and by age range) be as close as possible to the actual value.

Let us note b the birth rate (births / total population), e the emigration rate, i the immigration rate, d the death rate, p_0-4_ the proportion of the total population aged 0 to 4 and e_0-4_ (and similar notations) the share of the 0 to 4 years old population that emigrates over a 5-year period. Tweaked values will be noted b’, e’, i’ etc. We keep the overall population constant and minimize (d’_0-4_ – d_0-4_)^2^ + (e’_0-4_ – e_0-4_)^2^ + (i’_0-4_ – i_0-4_)^2^ and similar values for other age ranges. This leads to the following equations:

To keep the population constant:

b’ = p_0-4_

1 – p_5-9_ / p_0-4_ = d’_0-4_ + e’_0-4_ – i’_0-4_, …. 1 – p_80+_ / (p_75-79_ + p_80+_) = d’_75+_ + e’_75+_ – i’_75+_

To minimize the difference between the new rates and the actual rates:

3 (d’_0-4_ + e’_0-4_) = d_0-4_ + e_0-4_ + 2 (1 – p_5-9_ / p_0-4_) + 2 i_0-4_

…

3 (d’_75+_ + e’_75+_) = d_75+_ + e_75+_ + 2 (1 – p_80+_ / (p_75-79_ + p_80+_)) + 2 i_75+_

And:

2 d’_0-4_ + e’_0-4_ = d_0-4_ + (1 – p_5-9_ / p_0-4_) + i_0-4_

…

2 d’_75+_ + e’_75+_ = d_75+_ + (1 – p_80+_ / (p_75-79_ + p_80+_)) + i_75+_

The ‘actual’ (best estimates for the Piura region) and revised (values closest to reality while also maintaining the population constant) values of birth, “immigration” (movement into the village), “emigration” (movement out of the village) and death rates are given below.

Birth rates: 0-4 years old in 2016 represented 10.24% of the total 2011 Piura population. However, the 0-4 years old population in 2011 was 10.66% of the total population so, to ensure stability, 0-4 years old children 5 years later should represent 10.66% of the Piura population. To maintain the population structure, there should be a slightly higher birth rate than there actually is.

Immigration rates: to maintain the current population structure, there should be slightly more immigrants at the lowest ages (below 15 years old) but fewer immigrants at older ages than in reality. Overall, newcomers to Piura districts represented 9.3% of the Piura population, whereas the adjusted figure is 7.8%.

Emigration rates: to maintain the current population structure, there should be slightly fewer emigrants at the lowest ages (below 15 years old) but more numerous emigrants at older ages than in reality. Overall, an estimated 10.3% of the Piura population left between 2011 and 2016, and the adjusted emigration rate is 12.0%.

Death rates: to maintain the current population structure, there should be slightly fewer deaths at the lowest ages (below 15 years old) but more numerous deaths at older ages than in reality. Overall, an estimated 5.0% of the Piura population died between 2011 and 2016, and the adjusted death rate is 6.6%.

The difference between expectations and reality for total emigrants, immigrants, and deaths is 1.4 to 1.6 percentage points (in absolute value), whereas for births it is 0.4 percentage points. If we add births and immigrants to build “newcomers”, then we can write the estimated number of newcomers for Piura every 5 years (using the 2011 population as a basis):

With these new figures, demographics can be modelled as follows. At each time point and for each human:

The individual dies with a probability equal to the death rate for his age range (and one time step). If an individual dies, s/he is removed from the simulation and a new individual is created.

The individual (if not dead) emigrates with probability equal to the emigration rate for his age range (and one time step). Meanwhile, a new individual is created to replace the emigrant.

Creation of a new individual: the new individual is created as a newborn or immigrant in a given age range according to the share of all newcomers that are expected to be in that age range. If the new individual is an immigrant, s/he is either i) considered as coming from a “low risk” area with probability p_low_ (estimated at 61% based on currently available data) and from a “high risk” area otherwise.

Immigrants coming from low-risk areas are created with no taeniasis infection. The disease status of immigrants coming from a high-risk area is defined by i) taking a random individual in that age-range in any of the target villages, for all time points before the end of the burn-in period, ii) taking one or several “pictures” of the target villages around the end of the burn-in period and modelling the state of new immigrants from high-risk areas on a random individual in that or those “pictures”, choosing an individual in the appropriate age-range.

**S1 Table 6: Immigration into Piura: actual for 2011-2016 and revised values**

| Age range | 0-4 | 5-9 | 10-14 | 15-19 | 20-24 | 25-29 | 30-34 | 35-39 | 40-44 | 45-49 | 50-54 | 55-59 | 60-64 | 65-69 | 70-74 | 75+ |
| --- | --- | --- | --- | --- | --- | --- | --- | --- | --- | --- | --- | --- | --- | --- | --- | --- |
| Actual | 7.2% | 6.8% | 9.5% | 13.1% | 13.9% | 12.7% | 11.1% | 9.5% | 8.5% | 7.5% | 7.0% | 6.2% | 5.7% | 4.9% | 4.4% | 3.6% |
| Adjusted | 7.8% | 7.6% | 10.8% | 12.3% | 11.1% | 11.5% | 9.7% | 6.1% | 5.1% | 4.5% | 3.0% | 0.1% | 0.6% | 2.0% | 1.2% | 0.0% |

*Sources* [1–3] *and authors’ computations*

**S1 Table 7: Emigration from a Piura district: actual for 2011-2016 and revised values**

| Age range | 0-4 | 5-9 | 10-14 | 15-19 | 20-24 | 25-29 | 30-34 | 35-39 | 40-44 | 45-49 | 50-54 | 55-59 | 60-64 | 65-69 | 70-74 | 75+ |
| --- | --- | --- | --- | --- | --- | --- | --- | --- | --- | --- | --- | --- | --- | --- | --- | --- |
| Actual | 7.3% | 7.1% | 12.8% | 18.1% | 15.3% | 12.6% | 10.7% | 9.2% | 11.2% | 7.6% | 7.4% | 6.8% | 6.5% | 5.7% | 5.2% | 4.2% |
| Adjusted | 6.6% | 6.3% | 11.5% | 18.9% | 18.1% | 13.8% | 12.2% | 12.6% | 11.8% | 10.6% | 11.5% | 13.0% | 11.6% | 8.6% | 8.4% | 7.8% |

*Sources:* [1–3] *and authors’ computations*

**S1 Table 8: Deaths (plus emigration to another country): actual for 2011-2016 and revised values**

| Age range | 0-4 | 5-9 | 10-14 | 15-19 | 20-24 | 25-29 | 30-34 | 35-39 | 40-44 | 45-49 | 50-54 | 55-59 | 60-64 | 65-69 | 70-74 | 75+ |
| --- | --- | --- | --- | --- | --- | --- | --- | --- | --- | --- | --- | --- | --- | --- | --- | --- |
| Actual | 1.9% | 1.9% | 3.4% | 3.8% | 4.2% | 4.1% | 4.2% | 4.1% | 4.5% | 4.9% | 5.3% | 6.6% | 9.1% | 13.4% | 19.6% | 42.3% |
| Adjusted | 1.3% | 1.1% | 2.1% | 4.6% | 7.0% | 5.3% | 5.6% | 7.6% | 7.9% | 7.8% | 9.4% | 12.7% | 14.2% | 16.3% | 22.8% | 45.9% |

*Sources:* [1–3] *and authors’ computations*

**S1 Table 9: Newcomers – immigrants and births**

| Age range | 0-4 | 5-9 | 10-14 | 15-19 | 20-24 | 25-29 | 30-34 | 35-39 | 40-44 | 45-49 | 50-54 | 55-59 | 60-64 | 65-69 | 70-74 | 75+ |
| --- | --- | --- | --- | --- | --- | --- | --- | --- | --- | --- | --- | --- | --- | --- | --- | --- |
| Nb | 190271 | 14780 | 14498 | 20495 | 22825 | 18266 | 16279 | 12654 | 7356 | 5275 | 3995 | 2244 | 66 | 300 | 710 | 320 |
| Share | 57.6% | 4.5% | 4.4% | 6.2% | 6.9% | 5.5% | 4.9% | 3.8% | 2.2% | 1.6% | 1.2% | 0.7% | 0.0% | 0.1% | 0.2% | 0.1% |

*Number of individuals and share of all newcomers. Sources:* [3] *for Piura 2011 population and adjusted birth and immigration rates (cf. Table S 1.3)*

# References

1. United Nations, United Nation. World Population Prospects, Online Edition. Rev. 1. Rev 1; 2019.

2. Instituto Nacional de Estadística e Informática (INEI). Censos nacionales 2017: XII de poblacion, VII de vivienda y III de communidades indigenas, sistema de consulta de base de datos. Lima, Perú: INEI; 2017.

3. Ministerio de Salud - Oficina General de Tecnologías de la Información. Poblacion estimada por edades simples y grupos de edad, segun provincia y distrito, departamento de Piura. 2017.

4. Garcia HH, Gonzalez AE, Tsang VCW, O’Neal SE, Llanos-Zavalaga F, Gonzalvez G, et al. Elimination of Taenia solium transmission in northern Peru. New England Journal of Medicine. 2016;374: 2335–2344.

5. Beam M, Spencer AG, Atto R, Camizan R, Vilchez P, Muro C, et al. To really know the disease: Creating a participatory community education workshop about taenia solium focused on physical, economic, and epidemiologic evidence. American Journal of Tropical Medicine and Hygiene. 2019;100: 1490–1493.

6. Beam M, Spencer A, Fernandez L, Atto R, Muro C, Vilchez P, et al. Barriers to participation in a community-Based program to control transmission of Taenia solium in Peru. American Journal of Tropical Medicine and Hygiene. 2018;98: 1748–1754.

7. I.N.d.E.e.I.-. INEI/Perú E, editor. Perú Encuesta Demográfica y de Salud Familiar - ENDES 2012. ICF: LIMA; 2013.
