# Supplementary material for: Non-local validated parametrization of an agent-based model of local-scale Taenia solium transmission in North-West Peru
Source: PLoS One. 2022 Sep 27;17(9):e0275247. doi: 10.1371/journal.pone.0275247 (PMC9514638; doi:10.1371/journal.pone.0275247)
Supplement: S2 File — (DOCX) [file pone.0275247.s002.docx]

# Supporting information S2

ABM flow diagrams

#
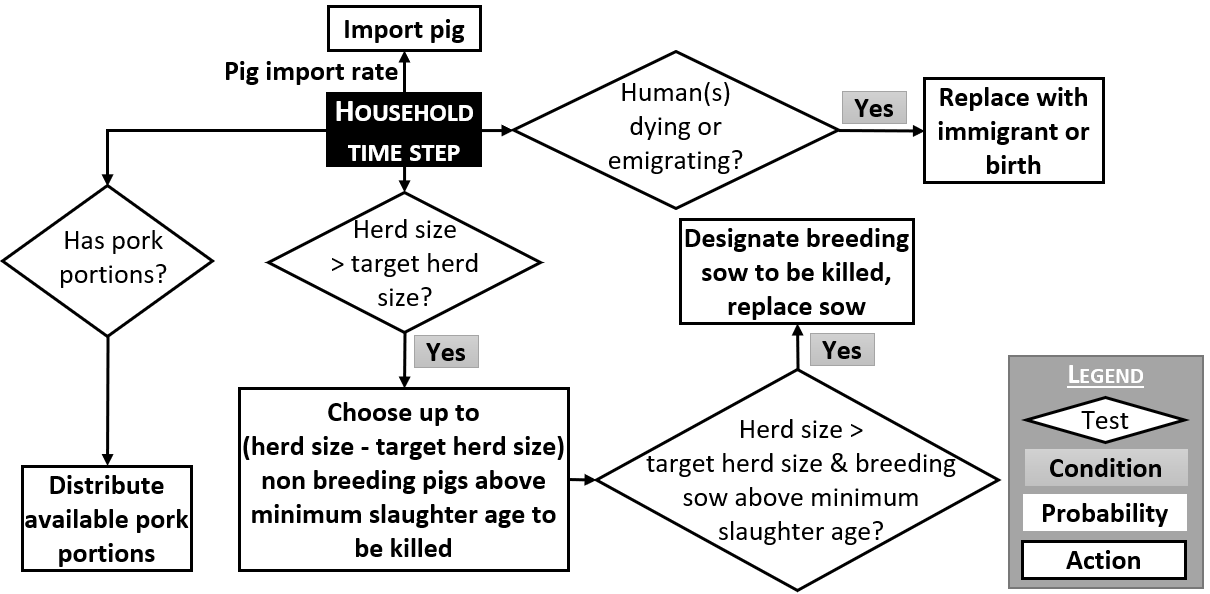


**S1 Fig 1: Household module flow chart.**


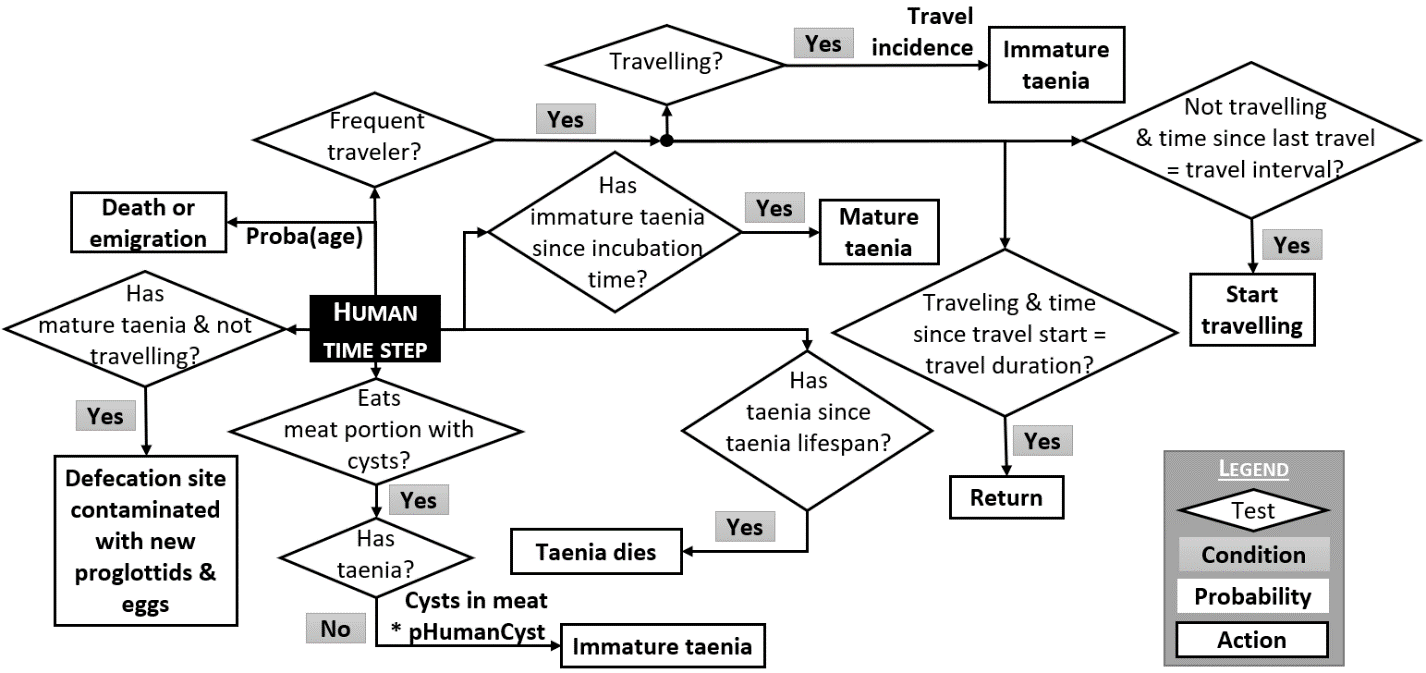


**S2 Fig2: Human module flow chart**. For simplicity reasons, travelers from outside of the village on short-term travel in the village have not been represented.

**S2 Fig3: Pig module flow chart.** The number of new cysts created after the exposure to contamination from proglottids are calculated using two realizations, $p_{\text{PO}}$ and $p_{\text{PH}}$, of a Poisson distribution of parameter pigProglotInf and cysts created after exposure to T. solium eggs are calculated using two realizations, $p_{\text{EO}}$ and $p_{\text{EH}}$, of a Poisson distribution of parameter pigEggsInf.
