# Supplementary material for: Non-local validated parametrization of an agent-based model of local-scale Taenia solium transmission in North-West Peru
Source: PLoS One. 2022 Sep 27;17(9):e0275247. doi: 10.1371/journal.pone.0275247 (PMC9514638; doi:10.1371/journal.pone.0275247)
Supplement: S3 File — (DOCX) [file pone.0275247.s003.docx]

# Supporting information 3

Additional data and figures

**S3 Table 1: Observed ABC target summary statistics.**

| **village** | **HT** | **PC** | **Number of necropsied pigs** |
| --- | --- | --- | --- |
| 515 | 0.024 | 0.33 | 17 |
| 566 | 0.010 | 0.15 | 16 |
| 567 | 0.020 | 0.16 | 44 |

|  | **village 515**  necro_i_ | **village 566**  necro_i_ | **village 567**  necro_i_ |
| --- | --- | --- | --- |
| 0 | 0,18 | 0,14 | 0,11 |
| 1 | 0 | 0 | 0,027 |
| 2 | 0 | 0 | 0 |
| 3 | 0,11 | 0 | 0,018 |
| 4 | 0,036 | 0 | 0 |
| 5 | 0 | 0 | 0 |

*The shares of pigs per number of cysts necro_i_, are normalized using the total number of pigs including non-infected pigs (zero cysts).*

**S3 Table2: variation ranges for calibration parameters that define the prior marginal distributions for the first ABC-SMC stage**

| **Calibration parameter** | **Lower limit** | **Upper limit** |
| --- | --- | --- |
| pHumanCyst | 0.0 | 5 10^-3^ |
| pigProglotInf (cysts) | 10 | 5000 |
| pigEggsInf (cysts) | 0 | 5000 |
| adherenceToLatrine | 0.1 | 0.95 |


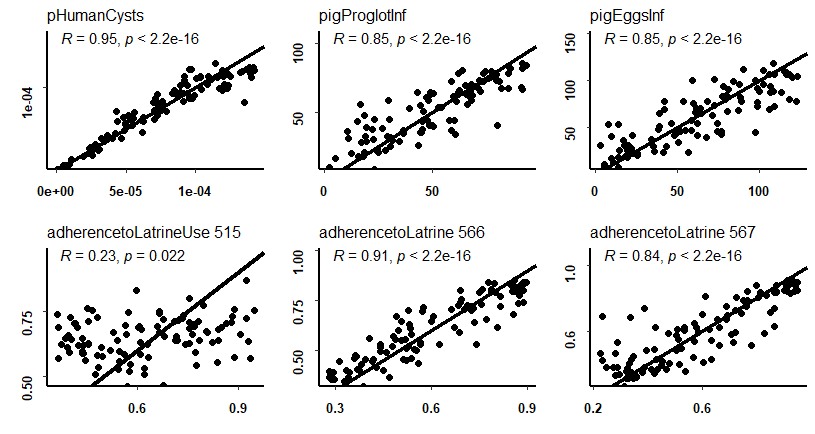


**S3 Fig 1: Cross validation necro setup.** Cross-validation for 100 randomly selected simulations from the necro calibration setup, third stage. Estimated parameter values are compared to true values for the 6 calibration parameters with the identity line plotted as reference. The correlation coefficient between estimated and true parameter values is shown for each parameter together with the corresponding p value.


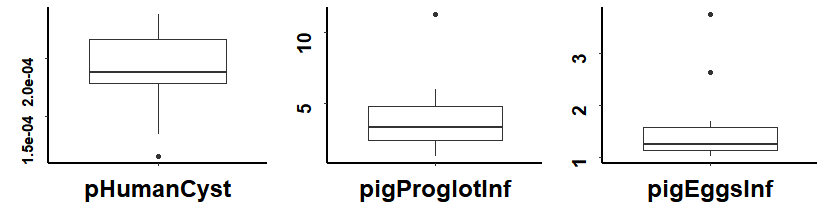


**S3 Fig 2:** **ABC posterior distribution, simplified calibration setup.** Horizontal bold lines indicate the posterior distribution median, boxes cover the parameter values above the first quartile and below the third quartile and vertical lines show maximum and minimum values. Black dots show outliers.
