# Supplementary material for: Non-local validated parametrization of an agent-based model of local-scale Taenia solium transmission in North-West Peru
Source: PLoS One. 2022 Sep 27;17(9):e0275247. doi: 10.1371/journal.pone.0275247 (PMC9514638; doi:10.1371/journal.pone.0275247)
Supplement: Attachment — Submitted filename: Review_tosubmit.pdf [file pone.0275247.s004.pdf]

Overall comments:

This paper represents potentially an important step in developing a transferrable *T. solium* transmission model, however I have several major comments/clarifications to raise, as the manuscript is hard to follow (particularly the results) in the present form, and some important questions regarding the methodology.

Intro

Comment 1: Can the authors expand on the following sentence, as its is not clear how this would be achieved (reducing the number of transmission parameters), and whether this is relevant to this paper (I don't think this was looked at in this paper)?

"*This can be addressed by streamlining steps representing parasite transmission in the ABM, thereby capturing the unknown probabilities in fewer transmission parameters*." (lines 89-91)

For example, in the first objective (outlined in 109-11), is the purpose also to streamline representation of parameters, and if so, this should be made clear in the objectives.

Comment 2: The following sentence is not very clear, please rephrase/restructure:

"This makes village-level calibration impractical converting it in a substantial barrier to the transferability of CystiAgent to most endemic settings." (lines 99 -101)

Comment 3: please can the author break up the sentence 114 – 119, as this sentence makes overall sense but is a little hard to follow.

Methods

Comment 1. What about seasonal differences in transmission (if influenced by the dry or 3-month rainy season indicated by the authors on lines 137-138). For example, Braae et al. 2014 found higher probability of free-ranging in the dry season compared to wet season and therefore different exposure risk (also possibly perturbing stable endemic transmission dynamics which most models currently assume). This study reflects the situation in Tanzania, a different endemic system, but may be relevant for this system.

Ref: Braae, U.C., Magnussen, P., Lekule, F. et al. Temporal fluctuations in the sero-prevalence of Taenia solium cysticercosis in pigs in Mbeya Region, Tanzania. Parasites Vectors 7, 574 (2014). https://doi.org/10.1186/s13071-014-0574-7

Comment 2. The authors mention in lines 139 – 140 that "latrines…are often of poor quality and readily accessible by humans". This seems like an important feature of this particular endemic system, so was information on latrine quality collected (alongside information on presence of latrine & adherence; line 158 & mapped on Fig 1) and incorporated into the model? Or do the authors assume that all latrines are of equally poor quality and therefore uniformly provide an exposure risk to free-roaming pigs?
If the latter (this seems the case, given lines 290-291 "*The level of contamination of the defecation site depends on the presence of a latrine in the tapeworm carrier household and on adherence to its use*"), this should be explicitly stated in the assumptions.

Comment 3. Regarding pig exposure (lines 183 – 188): Is the extent of pig exposure within a contaminated defecation site dependent on the duration of time spent in site, or assumed as soon as the pig agent enters a contaminated defecation site the pig will become exposed?

Comment 4. The authors state new human agents are "periodically introduced" into the simulation (lines 192 – 193), with the rich dataset acquired through this study, would it not be feasible (and more realistic) to accurately simulate immigration introductions (for example are there differential rates during dry vs rainy seasons)?

Comment 5. "cysts being distributed randomly to the pork portions" (lines 201-202) is a strong assumption to include, there is good knowledge now on the carcass distribution of cysts (see Chembensofu et al. 2017), so could the authors indicate whether they have considered modelling this (assuming pork portions can be from different parts of the pig carcass?).

The authors further state on lines 253 – 254 that "If the slaughtered pig is infected with T. solium, its cysts are distributed randomly to portions that are made of muscle, bones, and skin, but not to portions from entrails"; can the authors also explain here why cysts are not distributed to entrails (or a fuller description of what constitutes entrails would eb useful).

Ref: Chembensofu, M., Mwape, K.E., Van Damme, I. et al. Re-visiting the detection of porcine cysticercosis based on full carcass dissections of naturally Taenia solium infected pigs. Parasites Vectors 10, 572 (2017). https://doi.org/10.1186/s13071-017-2520-y

Comment 6. Please can the authors refer to the tables in the text where relevant and throughout, for example after describing the mean and standard deviation of the slaughter age (lines 228 – 231), referring to Table 2 here would improve clarity.

Comment 7. Given the large number of tables throughout the manuscript, I recommend condensing the tables, for example including in the same row, the overall parameters for slaughter age mean and standard deviation in Table 2 (and Table 4), rather than including two rows. The authors should try to do this across all tables to reduce the length of the methods.

Comment 8. After review of S1. Fig1. I am not clear where the decision process regarding sale or slaughter of pigs is included within the Household module flow chart. Indeed, it appears this is within the S2 Fig3: Pig module flow chart. Instead, which might cause some confusion when trying to match the description in the methods to these supplemental figures.

On a further note, S1 Fig 1 in the S2 word document should be S2 Fig 1? Can the authors check in detail throughout the supplementary to ensure there are no typos such as this please.

Comment 9. How valid is the assumption that "neighbouring areas have similar levels of T. solium transmission" regarding assigning the same probability of cysticercosis for imported pig agents (lines 240-242).

Comment 10. Can the authors provide more justification for why a maximum of one pork portion would be distributed to each human agent (lines 255 – 256), would it not be reasonable to expect different portions based on age and possible sex?

Comment 11. Furthermore, can the explain why pork portions are distributed more widely from the initial household (lines 256-260), are these pork portions sold to the other

households, or given freely? If sold, is there a probability associated with the ability to pay for the recipient household, or is this effectively captured in the *pigimportRateHousehold* parameter (Table 2)?

Probabilities in Table 2??? – should they be represented by a prob distribution w/ parameters?

Comment 12. Should there not be a decay rate associated with proglottids (or eggs remaining within these proglottids) in the environment (295-298; "*When the tapeworm dies, the site is no longer contaminated with proglottids, but eggs remain present until they deteriorate and are no longer infective to pigs*". It appears at the moment as though proglottids (or eggs within) instantly disappear upon death of the adult worm?

Comment 13. How was the tolerance threshold of 0.015% chosen (line 399)?

Comment 14. On line 451, the authors write "We assessed the fit of calibration parameters using cross-validation (19). The cross-calibration method"; are these methods the same, or should the second by changed from cross-calibration to cross-validation (i.e., I don't think cross-calibration has been mentioned before in "Calibration distinguishing local from non-local parameters" section)

Minor (methods) comments:

Comment 1. Infrastructure instead of infrastructures (line 145)

Comment 2. A reference is missing on line 148, 244; "Error! Reference source not found!"

Comment 3. The wording in the following sentence could be improved for clarity, and Significative effects should be rephrased to significant effect (line 154):

"*Data from the 5 intervention villages, in which however, as showed in the original study (10), interventions produced no significative effects on observed HT and PC prevalence, were then used to validate the final calibration process*".

Comment 4. Line 178, this could be reworded to improve clarity, such as "with a circular defecation site area around its household"

Comment 5. Definitely should be definitively on line 191.

Comment 6. averages should be average (line 241)

Comment 7. The authors explain the calibration has been limited to "those parameters that may be relatively invariant among villages", meaning the non-local calibration parameters (in tables 3-4) and while there is a description of "distinguishing local from non-local parameters" in the statistical methods from line 419, it would be useful if earlier in the methods the authors explicitly state that there are both local (e.g., adherence to latrines in Table 3) and non-local parameters

Comment 8. Can the authors provide references (or an indication that the work is from local survey work, not published?) for travelProp, travelFreq and travelDuration parameters in Table 3 please?

Comment 9. The sentence on line 367 to 370 regarding development of cysts is not clear, please can the authors break this into two sentences and re-think the wording to improve

clarity. This will also help to improve understanding of sentence 373 to 374 regarding parameters definitions of *pigProglotInf* and *pigEggsInf*.

Comment 10. Please can the authors write lognormal mean and lognormal SD under notes and references for *homeRangeMean* and *homRangeSd* in table 4. Please also change Share of time to Proportion of time for *pigPHomeArea* to keep consistent with the text.

Results (pg.27)
Overall, some error and not easy to follow, so I would encourage the authors to think carefully about how to better present these results. There are some errors (in tables) and missing references/ lack of text to support inclusion of tables (e.g., Table 6) that make the results difficult to digest. In the results, similar to previous sections, there are a number of "(Error! Reference source not found.)" insertions, which additionally makes the results section a challenge to understand. Specific comments follow:

Comment 1. Can the authors include y-axis labels for figure 4 please on the plots.

Comment 2. I am not entirely sure what Figure 4 is showing, is this the range from many different simulations obtaining different posterior distributions, or is the box and whisker plot for each parameter showing a single posterior distribution (with the median, IQR etc for that single posterior distribution). If the latter, it would be good to also show the posterior distribution (probability density) plots in addition.

Comment 3. The authors also state that there is a "remarkable narrowing of the posterior marginal distributions for the non-local calibration parameters of the noNecro calibration setup" (Figure 4 panel b) compared to the prior marginal distributions (supplementary information 3); I am not clear what prior marginal distributions the authors are comparing to in the supplementary file 3 – are the authors referring to S3 Table 2; if so, please make clear in the main text?

Comment 4. There seems to be some errors regarding the Relative Error (RE) values in Table 5, for example, for the HT RE in 566, for the necro setup, this is 86% ((0.0097-0.0014)/0.0097 = 0.8556), but noNecro is also 86%, however for noNecro RE calculation, (0.0097-0.018)/0.018 = -0.4611111 (-46%), unless there is some misunderstanding here?

Comment 5. "*This is reflected by the lower values obtained when the necro distance Pnecro is calculated using the best runs produced by the necro calibration setup as compared to the best run of the noNecro setup*" (lines 506 – 508). Not clear what lower values mean here, the distance between observed and simulated?

Comment 6. There is no reference to Table 6, should this be in line 508? If this is the case, Table 6 is quite complex so the authors should explicitly indicate which elements of lines 506-508 (if these indeed refer to Table 6) refer to specific findings in Table 6, or include further text to explain this table.

Comment 7. Please can the authors include x and y-axis lables, for example, I am assuming that the y-axis is the true values and the x-axis is the estimated parameter values for simulations (and it is not clear in the figure legend lines 560 – 563)? Can the authors also explicitly include reference to Figure 6 in the Cross-validation results section (lines 544-557).

Comment 8. The Cross validation necro setup scatter plots S3: Fig 1 seem to suggest that the necro setup performs far better a reproducing the local "*adherenceToLatrine*" parameter compared to the noNecro (especially for villages 566 and 567), is there a reason why these results were not included in the main results?

Comment 9. Can the authors include the observed values again for each village in Table 7, or at least say in the Table legend that the RE are calculated with observed prevalence's from table 5, and the corresponding formula.

Comment 10. I would suggest making really clear, in the opening of the "Simplification of the Calibration" results section (page 31.) that simplified calibraiton does not calibrate the local parameter (adherence to latrines) therefore does not include the NoNecro and Necro setup (so the simplified calibration results can be compared to NoNecro and Necro setups). This will save the reader from going back to the methods to make the connection that NoNecro and Necro require the adherence to latrine parameter.

Comment 11. While the authors highlight that the orders of magnitude are similar for the non-local parameters in the noNecro and simplified setups, which is true (Table 8), I am not convinced by the statement that the "*simplified model calibration is able to estimate precisely the calibration parameters*" (lines 574 – 575) – while they are not far off, particularly for the pigProglotIn probability there is quite a difference i.e. 3.86 to 11.23, so I think this statement is too strong to make. Are there uncertainty estimates available from the fitting procedure for each parameter, to then assess whether there is overlap in these uncertainty ranges (95% credible intervals) to make the comparison more valid? There is a reference missing on page 574, which might refer to Figure 7, in which case a stronger argument can be made for this statement?

Comment 12. The authors state "The result of the simulations demonstrates that the parametrization obtained from the global calibration procedure presented here can be effectively exported to similar villages without any additional adjustment." (lines 595 – 594) and while the average RE might similar, the variation in RE (particularly for higher REs) seems greater for the validated results to intervention villages (with max errors of 75% for HT (validated) compared to 45% for HT (calibrated) and 117% for PC (validated) compared to 75% for PC (calibrated)). I think the authors need to caveat the above statement, especially if they are interested in transferring the model to look at individual villages in a different setting. Is there for example a threshold difference in average RE that the authors would then the globally calibrated model would not be transferrable to another endemic setting?

Discussion

Overall, this is a good discussion analysing the strengths and current limitations of the model. I think, as mentioned in *Results comment 11.*, indicating whether there was overlap in credible intervals best-fitting non-local parameter (between noNecro and simplified) for example would make statements around transferability more valid, especially as the authors argue that the RE metric should be interpreted with caution (in terms of assessing how transferrable the model is, especially given the extremes in some of the REs between the calibrated vs. validated simplified model).

The authors mention that the "resulting validation process is based on gold standard methods", which is certainly true for necropsy, although there is limited detail on the diagnostic used for taeniasis (is this just copro-antigen assessment for example, which has substantial limitations)?
